# Characteristics of patients with metastatic renal cell carcinoma who do not respond to axitinib treatment

**DOI:** 10.1007/s10147-025-02715-3

**Published:** 2025-02-14

**Authors:** Kojiro Ohba, Takahiro Osawa, Takahiro Kojima, Tomohiko Hara, Mikio Sugimoto, Masatoshi Eto, Keita Minami, Yasutomo Nakai, Kosuke Ueda, Sei Naito, Norio Nonomura, Sachiyo Murai, Hiroyuki Nishiyama, Hiromi Nakanishi, Yuta Mukae, Kensuke Mitsunari, Tomohiro Matsuo, Ryoichi Imamura, Nobuo Shinohara

**Affiliations:** 1 Department of Urology and Renal Transplantation, Nagasaki University Hospital, 1-7-1 Sakamoto, Nagasaki, 852-8501 Japan; 2 Department of Urology, Hokkaido University Hospital, Sapporo, Japan; 3 Department of Urology, Aichi Cancer Center, Nagoya, Japan; 4 Office of Research Administration, Center for Regulatory Science, Pharmaceuticals and Medical Devices Agency, Tokyo, Japan; 5 Department of Urology, Kagawa University, Kagawa, Japan; 6 Department of Urology, Kyushu University, Fukuoka, Japan; 7 Department of Urology, Sapporo City General Hospital, Sapporo, Japan; 8 Department of Urology, Osaka International Cancer Institute, Osaka, Japan; 9 Department of Urology, Kurume University Hospital, Kurume, Japan; 10 Department of Urology, Yamagata University, Yamagata, Japan; 11 Department of Urology, Osaka University Hospital, Osaka, Japan; 12 Department of Urology, University of Tsukuba Hospital, Tsukuba, Japan; 13 Department of Urology, Kushiro Rosai Hospital, Kushiro, Japan

**Keywords:** Axitinib, Metastatic renal cell carcinoma, Disease progression, Tyrosine kinase inhibitor, Nivolumab

## Abstract

**Background:**

Axitinib is a widely used tyrosine kinase inhibitor (TKI) in metastatic renal cell carcinoma (mRCC) treatment. Here, we analyzed the characteristics of patients who did not respond to axitinib and evaluated alternative options for their treatment.

**Methods:**

We retrospectively analyzed data for 449 patients with mRCC who were administered axitinib following another TKI as initial therapy. Patients with progressive disease (PD) at their first assessment were defined as showing early-PD. We analyzed the characteristics of patients at risk of early-PD and evaluated the relationship between the treatment following axitinib and their prognosis.

**Results:**

Early-PD was diagnosed in 102 patients, and was more common in those who had not undergone nephrectomy (*p* < 0.001), those treated with a TKI for a short period (*p* < 0.001), and those in the International Metastatic Renal Cell Carcinoma Database Consortium (IMDC) poor risk category for mRCC (*p* < 0.001). Multivariate analysis showed that these were independent risk factors for early-PD (all *p* < 0.001). Of those with early-PD, 52 changed to next-line treatment. The progression-free survival periods were 5.5 (95% confidence interval (CI) 2.4–8.6) months for patients administered TKIs, 4.2 (95% CI 0.3–8.1) months for those on nivolumab, and 2.2 (1.8–2.6) months for those on mammalian target of rapamycin inhibitors (*p* = 0.030).

**Conclusion:**

Patients who have not undergone nephrectomy, those previously treated with another TKI for a short period, and those in the IMDC poor risk category are more likely to experience early-PD when taking axitinib. Furthermore, TKIs are the best treatment for patients with early-PD who have previously been administered axitinib.

## Introduction

Over 430,000 new diagnoses of renal cell carcinoma are made per year worldwide, and these are accompanied by approximately 180,000 deaths [1]. Approximately 20–40% of such patients develop metastatic renal cell carcinoma (mRCC) [2]. The standard first-line treatment of mRCC comprises an immuno-oncology (IO) drug in combination with another IO drug or a vascular endothelial growth factor receptor-tyrosine kinase inhibitor (VEGFR-TKI) [3–7]. However, most patients require further rounds of treatment, and the drugs used for these are almost always TKIs. Thus, TKIs remain one of the most important treatment options, even during the IO era, and therefore, their use is worthy of analysis.

Axitinib was approved as a second-line or later treatment for mRCC on the basis of the results of a large randomized phase 3 trial [8]. It is a potent and selective inhibitor of VEGFR 1, 2, and 3 at low doses [8], and whereas other TKIs inhibit PDGFR, KIT, RAF, FLT-3, and other molecules, axitinib does not, and it is therefore associated with relatively mild adverse events. Axitinib is used in combination with IO drugs as a first-line treatment, but is also frequently used as second-line or later treatment, making it one of the most widely used TKIs in patients with mRCC. However, because there are patients in whom axitinib is ineffective, and given that a number of other TKIs are available, it is important to be able to predict its efficacy and the course of disease after a failure of treatment using this drug. Nevertheless, there have been very few studies of these issues to date.

In the present study, we compared the data collected for patients in whom axitinib was or was not effective, and identified the factors associated with this difference. We also evaluated the treatment options and outcomes of patients following axitinib treatment, and specifically those of patients who experienced early progression of disease (early-PD) of the disease while undergoing axitinib treatment.

## Patients and methods

A retrospective multicenter study was conducted at 36 institutions to evaluate the treatment outcomes of 590 patients with mRCC who were administered a VEGFR-TKI as first-line therapy, followed by axitinib, between January 2012 and February 2019 [9]. The target of this study is representative of mRCC in this period with TKI as the 1st line. Of these patients, those for whom a pathologic diagnosis was not available, those who had been treated with axitinib for < 4 weeks, those who were followed for < 3 months, and those with missing data were excluded, after which 449 remained to be studied. The Institutional Review Board of Hokkaido University Hospital (the principal institution) approved the study protocol (approval number 018–0003), as did the review boards of each of the participating hospitals. The patients studied received routine clinical care at the discretion of each physician, including with respect to the use of axitinib and subsequent therapy.

The International Metastatic Renal Cell Carcinoma Database Consortium (IMDC) risk classification and metastatic regions as baseline data were assessed immediately before axitinib administration [10]. The response of the tumor to therapy was classified as a complete response (CR), a partial response (PR), stable disease (SD), or progressive disease (PD), according to the Response Evaluation Criteria in Solid Tumors (RESIST), version 1.1 [11]. Those patients who showed PD at the time of the initial assessment of efficacy were defined as showing the early-PD. The overall duration of survival (OS) was defined as the period of time from the start of axitinib therapy to all-cause death or the date of the final follow-up appointment, and progression-free survival (PFS) was defined as the period between the start of axitinib therapy and the time of the first documentation of progression or death.

### Statistical analyses

Continuous data are presented as medians and ranges, and categorical data are presented as counts and percentages. The Mann–Whitney U and chi-square tests were used to compare continuous and categorical datasets, respectively. Kaplan–Meier analysis was used to calculate PFS and OS, and comparisons were made using the log-rank test.

Multiple logistic regression analysis was used to identify parameters that were associated with early-PD, and the risks are expressed as odds ratios and 95% confidence intervals (CIs). Parameters with *P* < 0.05 on univariate analysis were included in the multivariate analysis. A two-tailed *P* < 0.05 was accepted as indicating statistical significance. Statistical analyses were performed using SPSS Statistics for Windows (version 19.0; IBM Corp., Armonk, NY, USA).

## Results

### Characteristics of the patients

The characteristics of the patients included in the study are listed in Table 1. The median follow-up period was 18.0 months (range 3–72 months), the median duration of first-line treatment with a TKI was 7.0 months, and most of the patients were administered axitinib as their second-line treatment. The type of TKI being administered immediately before axitinib administration was sorafenib in 97 patients, sunitinib in 313 patients, and pazopanib in 39 patients. Of the patients, 12.7%, 66.6%, and 20.7% were classified into favorable, intermediate, and poor risk categories, respectively, according to the IMDC risk classification. The most common sites of metastasis were the lungs (70.4%), bones (31.6%), and lymph nodes (28.5%). The median PFS (95% CI) during axitinib treatment was 10.5 (9.3–11.7) months. Of the 449 patients, 46 continued to respond to treatment and 246 underwent alternative treatment.

**Table 1:** Characteristics of the patients treated with axitinib

	*N* = 449	
Age (y.o), median (IQR)	67	(61–74)
Gender, *n* (%)		
Male	344	(76.6%)
Female	105	(23.4%)
Prior nephrectomy, *n* (%)	405	(90.2%)
Histology, *n* (%)		
Clear cell carcinoma	400	(89.1%)
Non-clear cell carcinoma	49	(10.9%)
Number of previous lines before axitinib, *n* (%)		
1 line	393	(87.5%)
2 lines	56	(12.5%)
Pre-treated TKI (1st / 2nd), *n* (%)		
Sorafenib	107/18	
Sunitinib	309/30	
Pazopanib	33/8	
Duration of 1st line TKI (months), median (IQR)	7.0	(3.0–17.0)
IMDC risk group, *n* (%)		
Favorable	57	(12.7%)
Intermediate	299	(66.6%)
Poor	93	(20.7%)
Metastatic site, *n* (%)		
Lung	316	(70.4%)
Bone	142	(31.6%)
Lymph node	128	(28.5%)
Liver	72	(16.0%)
Pancreas	34	(7.6%)
Brain	23	(5.1%)

### Evaluation of the patients who progressed to early-PD

Of the 449 patients studied, 102 were diagnosed with early-PD. The median PFS (95% CI) of these patients was 2.9 (2.6–3.2) months, and that for patients who did not show PD was 14.5 (12.2–16.8) months (*p* < 0.001). Diagnoses of early-PD were significantly more common in patients who had not undergone nephrectomy (*p* < 0.001), those who had previously been treated with a TKI for a short period of time (*p* < 0.001), and those in the IMDC poor risk category (*p* < 0.001) (Table 2). There were no differences of responses among metastatic regions. Multivariate analysis showed that not undergoing nephrectomy, a short period of treatment with the previous TKI, and being in the IMDC poor risk group were found to be independent risk factors for early-PD (all *p* < 0.001). In contrast, the type of TKI administered immediately before axitinib administration was not associated with the risk of early-PD (*p* = 0.405).

**Table 2:** Results of the univariate and multivariate logistic regression analysis to identify factors affecting early-PD in patients treated with axitinib

	Univariate analysis			Multivariate analysis		
	OR	95% CI	*P*	OR	95% CI	*P*
Age (y.o)	0.977	0.944–1.010	0.169			
Non-clear cell carcinoma	1.358	0.689–2.677	0.363			
Previous 2 TKIs (vs 1 TKI)	0.765	0.371–1.577	0.603			
Total duration of prior TKI < 7 months	1.832	1.161–2.890	0.011	1.357	0.833–2.212	0.221
Non-prior nephrectomy	4.899	2.575–9.319	0.000	3.704	1.873–7.299	0.000
IMDC poor risk	3.959	2.404–6.510	0.000	3.268	1.938–5.495	0.000
Bone metastasis	1.293	0.806–2.073	0.324			
Liver metastasis	1.257	0.698–2.264	0.438			
Brain metastasis	1.633	0.652–4.091	0.301			

### Outcomes of axitinib treatment and findings for patients being administered axitinib who showed early-PD.

**Fig. 1:**
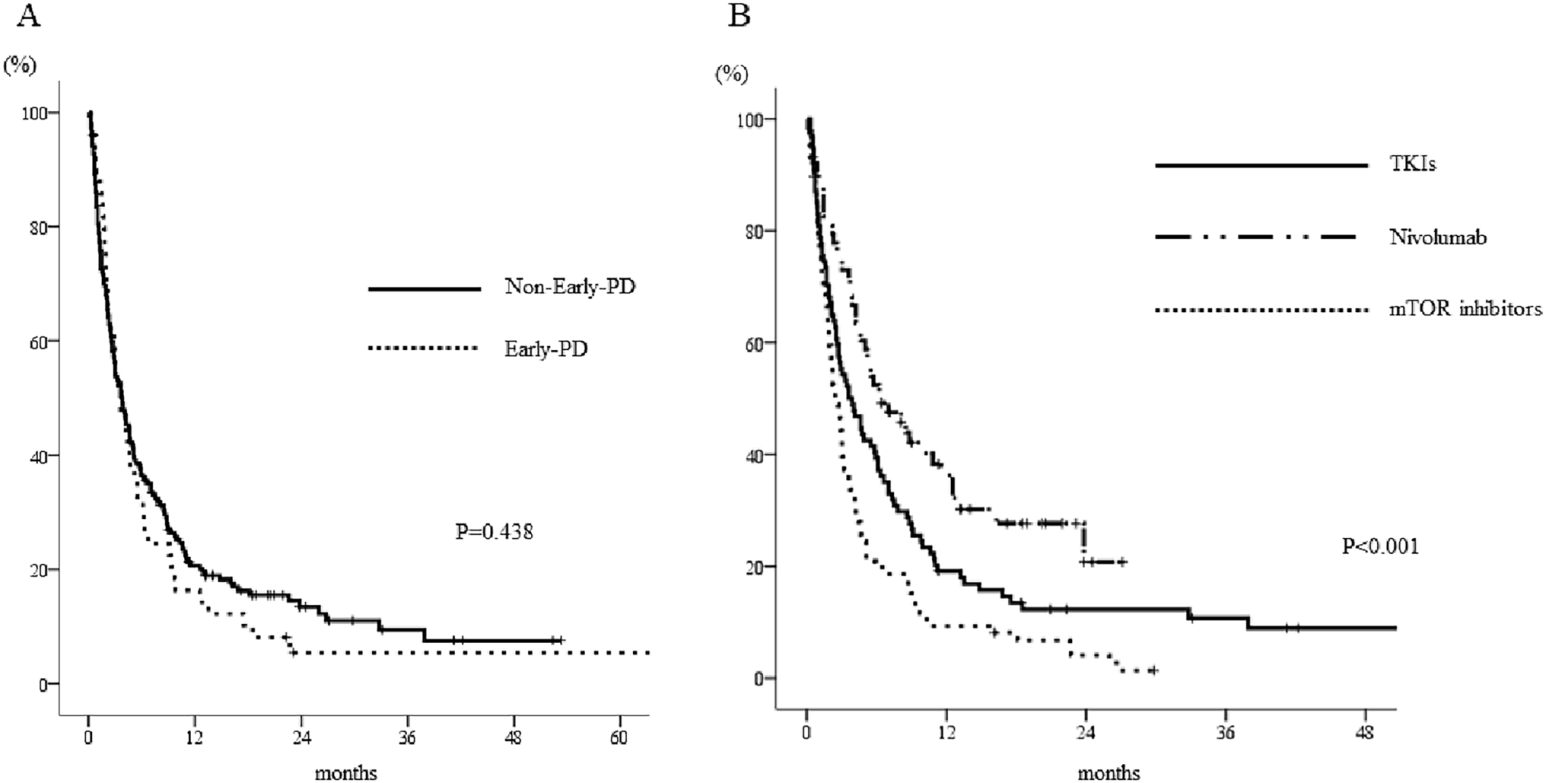
Kaplan–Meier curves for the PFSs of patients who underwent alternative therapy following axitinib treatment. **a** There was no significant difference in the median PFS of the patients who did or did not show early-PD (*p* = 0.438). **b** There was a significant difference in the median PFSs of the patients according to the therapeutic regimen used (*p* < 0.001). The log-rank test was used for statistical analysis. *TKIs*, tyrosine kinase inhibitors, *mTOR* mammalian target of rapamycin, *PFS* progression-free survival

**Fig. 2:**
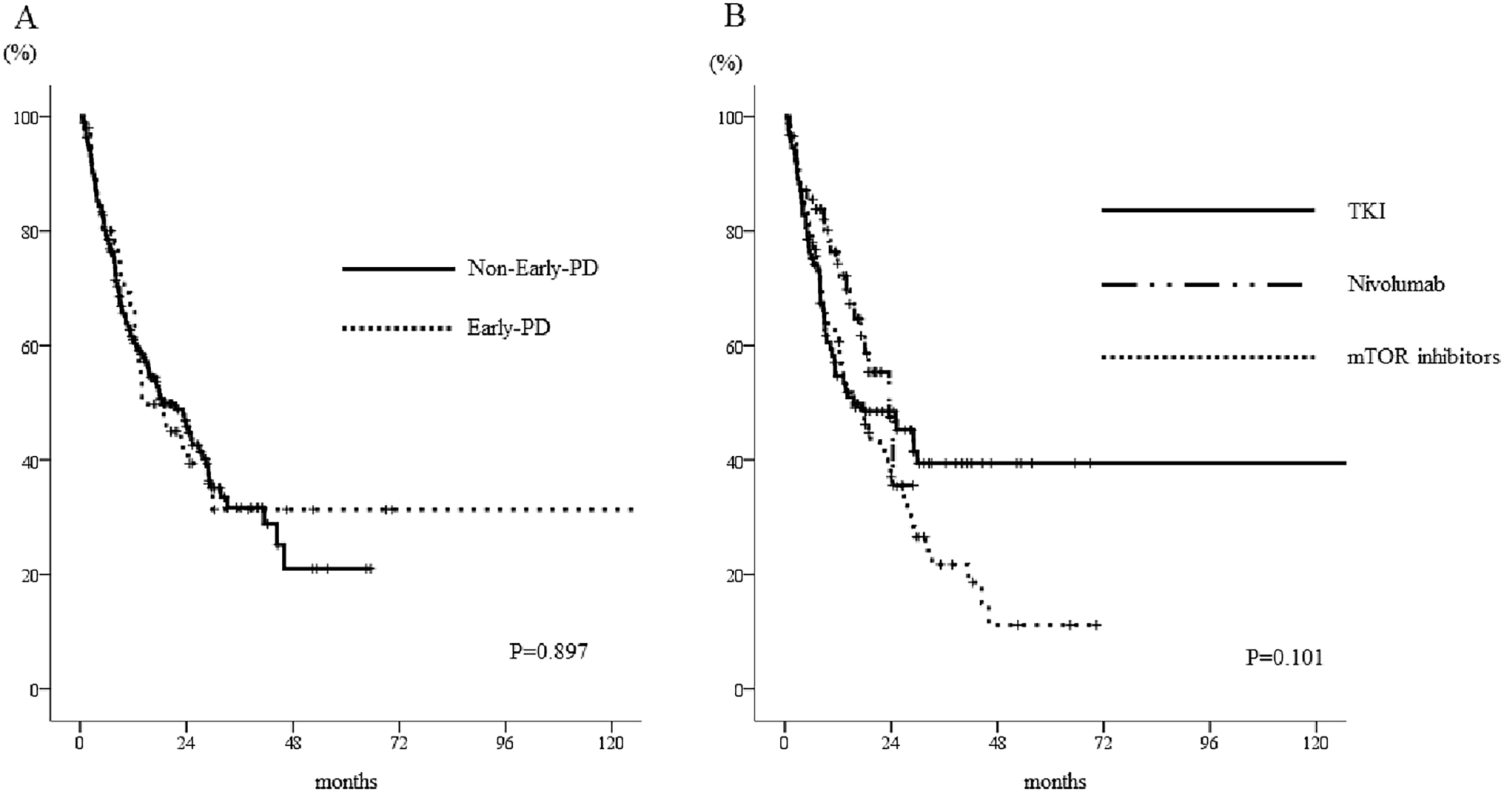
Kaplan–Meier curves for the OSs of patients who underwent alternative therapy following axitinib. There were no differences a) in the OS of patients who did or did not show early-PD after axitinib treatment (*p* = 0.897) or b) in those in whom differing regimens were used (*p* = 0.101). The log-rank test was used for statistical analysis. *TKIs* tyrosine kinase inhibitors, *mTOR* mammalian target of rapamycin, *OS* overall survival

When we analyzed the outcomes of the 246 patients who underwent a further treatment after axitinib treatment, we found no significant difference in the PFSs of the early-PD group (median 3.6 months, 95% CI 2.4–4.8) and the no-early-PD group (median 3.7 months, 95% CI 2.7–4.7) (*p* = 0.438, Fig. 1A). There was a significant difference in the PFSs of the patients who were on differing treatment regimens: 6.4 (95% CI 3.0–9.8) months for nivolumab, 3.6 (95% CI 2.0–5.2) months for TKIs, and 2.5 (1.8–3.2) months for mammalian target of rapamycin (mTOR) inhibitors (*p* < 0.001, Fig. 1B). However, there were no differences in the OSs of the patients after axitinib therapy according to their early-PD status or the regimen used (*p* = 0.897 and 0.101, respectively; Fig. 2A, B).

**Fig. 3:**
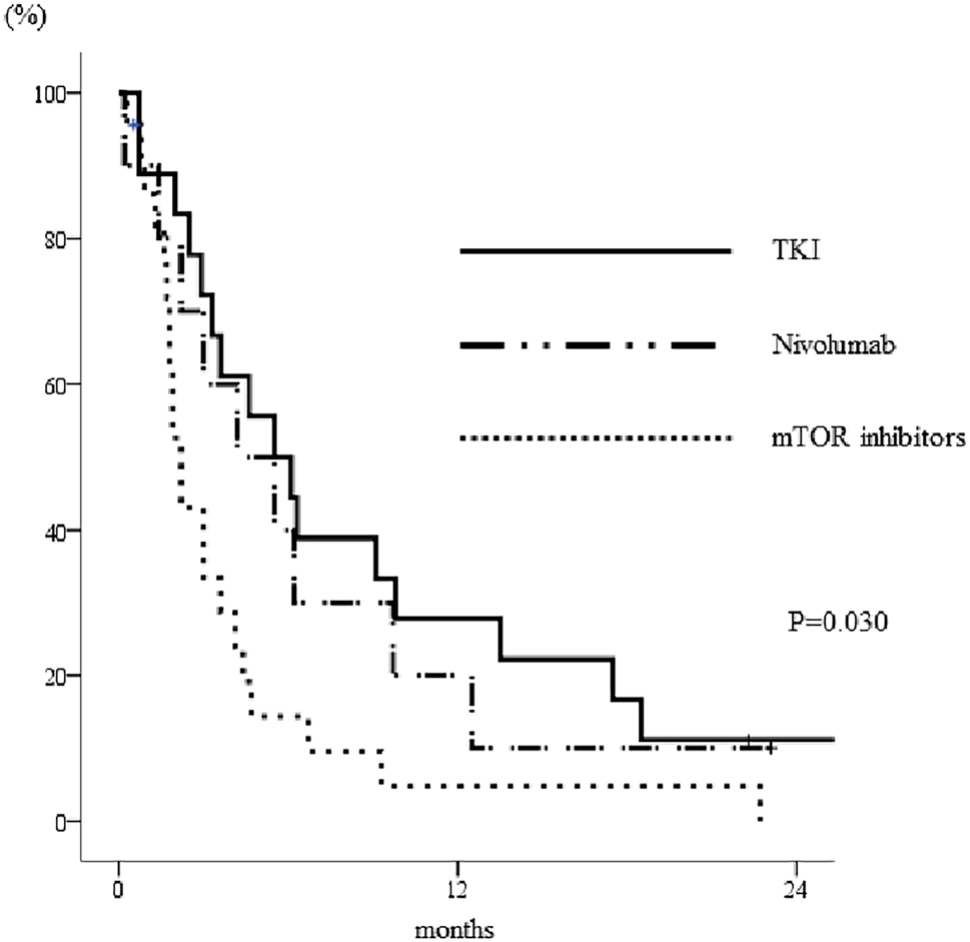
Kaplan–Meier curves for the PFSs of patients with early-PD who underwent alternative therapy following axitinib treatment. Patients treated with a TKI after axitinib therapy had superior outcomes to those treated with nivolumab or an mTOR inhibitor (*p* = 0.030). The log-rank test was used for statistical analysis. *TKIs* tyrosine kinase inhibitors, *mTOR* mammalian target of rapamycin, *PFS* progression-free survival

**Fig. 4:**
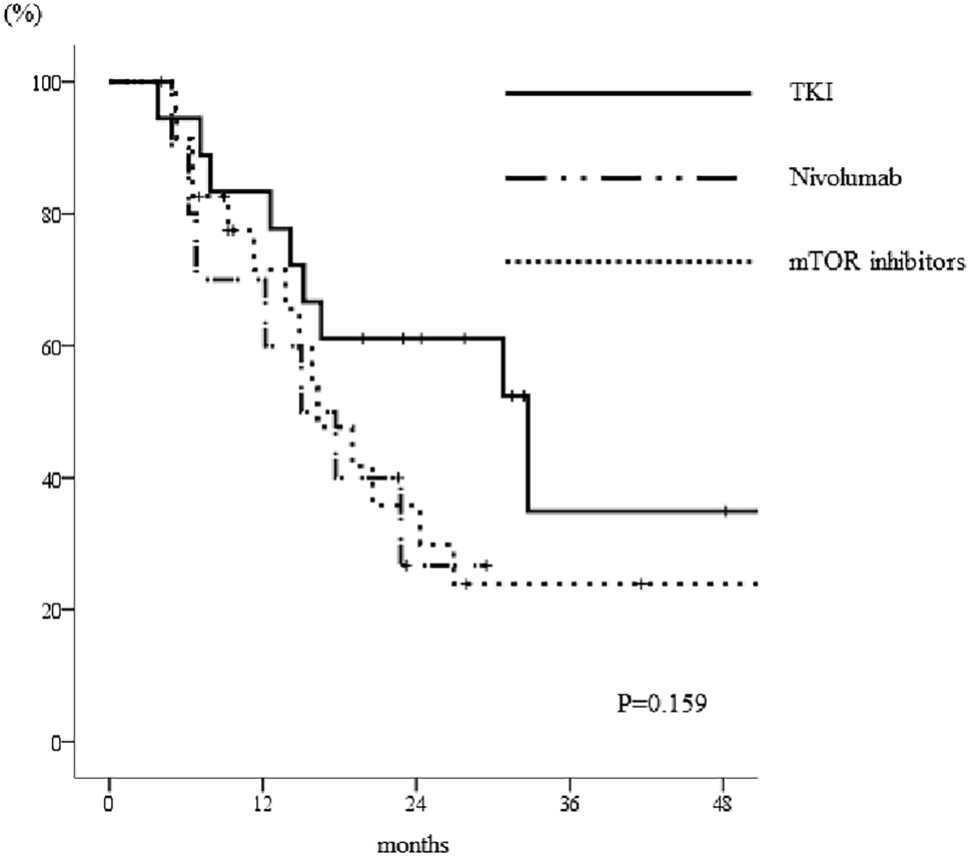
Kaplan–Meier curves for the OSs of patients with early-PD who underwent alternative therapy following axitinib treatment. Patients treated with a TKI tended to have longer OSs than those treated with nivolumab or an mTOR inhibitor, although these differences were not significant (*p* = 0.159). The log-rank test was used for statistical analysis. *TKIs* tyrosine kinase inhibitors, *mTOR* mammalian target of rapamycin, *OS* overall survival

Of the 102 patients undergoing axitinib who developed early-PD, 52 underwent next-line treatment and 50 received the best supportive care available. There was a significant difference in the PFSs of patients according to the regimen used for patients with early-PD following axitinib therapy: 5.5 (95% CI 2.4–8.6) months for TKIs, 4.2 (95% CI 0.3–8.1) months for nivolumab, and 2.2 (1.8–2.6) months for mTOR inhibitors (*p* = 0.030, Fig. 3). Patients with early-PD who were treated with a TKI tended to show better OS (median 32.7 months, 95% CI 12.7–52.7) than those treated with nivolumab (median 15.0 months, 95% CI 6.5–23.5) or an mTOR inhibitor (median 16.3 months, 95% CI 10.9–21.6), although this difference was not significant (Fig. 4, *p* = 0.159).

## Discussion

Axitinib is used as a systemic therapy for mRCC, typically as a second-line or later treatment when previous therapeutic regimens have failed or are no longer suitable. Therefore, early-PD after axitinib treatment is frequently associated with mortality. Therefore, it is crucial to identify patients for whom axitinib may be ineffective before treatment. In the present study, the following were identified to be independent predictors of early-PD during axitinib treatment: (1) a lack of nephrectomy, (2) brief previous TKI therapy, and (3) belonging to the IMDC poor risk category.

Since the administration of TKIs began, nephrectomy has been reported to improve the prognosis of mRCC [12–19]. However, the CARMENA trial, a randomized controlled trial that evaluated the effect of nephrectomy on the prognosis of mRCC, did not demonstrate a survival benefit [20]. However, these results may not be definitive, because the OS of the patients after nephrectomy was only 13.9 months, suggesting that the included patients might not have been suitable candidates for the procedure, because of aggressive disease. The necessity for nephrectomy remains a topic for debate, because it is thought that nephrectomy may improve the prognosis of patients by removing TKI-resistant clones or alleviating cancer-related symptoms related to cytokine release. However, in patients in whom nephrectomy is difficult to perform, the disease may be more aggressive, and therefore, TKI therapy might be less effective. Given that many studies have shown that patients who undergo nephrectomy have a better prognosis, those who undergo the procedure may respond better to TKI therapy, including with axitinib.

The AXIS trial showed that patients underwent a shorter period of axitinib treatment following sunitinib than interferon therapy (4.8 months *vs*. 12.1 months) [8], implying that the previous treatment affects the duration of axitinib therapy. In fact, a longer duration of previous TKI therapy has been reported to be an independent prognostic factor for mRCC [21]. Transcriptomic and epigenomic sequencing have also shown that some types of renal cancer are less responsive to TKI treatment than others [22], and such cancers may retain this property during subsequent treatments, which may explain the early-PD of disease associated with axitinib administration.

The IMDC risk classification is a model that is used to predict the treatment outcomes of patients during TKI administration. The patients in the present study were treated with axitinib as their second- or third-line therapy, and it has been reported that the efficacy of TKI treatment does not significantly vary according to the order in which treatments are administered [23]. Therefore, it is reasonable to use the IMDC risk classification for the prediction of TKI treatment efficacy, regardless of the order of treatment, and this may explain why belonging to the IMDC poor risk category is predictive of early-PD. For patients at risk of early-PD while undergoing axitinib therapy, the use of alternative treatments such as cabozantinib, which has been shown to be associated with favorable outcomes after TKI therapy, might be considered [24].

Interestingly, patients who exhibited early-PD and were subsequently treated with another TKI had better PFSs than those treated with nivolumab or an mTOR inhibitor. Among the patients who underwent further treatment after axitinib treatment, those who were treated with nivolumab had better PFSs than those who were treated with TKIs or mTOR inhibitors. Although the CM025 trial demonstrated the superiority of the use of nivolumab to that of mTOR inhibitors following TKI treatment, it did not include a comparison with the use of TKI therapy [25]. Furthermore, there was no mention regarding the potential relationship between the efficacy of nivolumab and that of the prior TKI. In the present study, we have shown that the use of nivolumab is associated with a better PFS than the use of a second TKI following the administration of a first TKI. However, the opposite trend was identified in those who showed early-PD. The reason why patients treated with TKIs had better outcomes than those treated with IO drugs when showing early-PD following axitinib therapy are unclear. This suggests that TKIs may remain an important treatment option for patients who are refractory to axitinib therapy, potentially because they inhibit multiple kinases. Currently, immune checkpoint inhibitors are commonly used as first-line therapies for mRCC, often in combination with TKIs, such as cabozantinib or lenvatinib, but these were not administered in the present study. Therefore, we do not know whether the outcomes of axitinib treatment would be similar using these treatment protocols. Furthermore, there were no differences in the OSs of patients who did or did not show early-PD or in those who underwent differing therapeutic regimens after axitinib treatment. TKIs are frequently used as a second or subsequent-line treatment, indicating that they can still play a significant role in the IO era and that their use as a therapeutic option should be re-evaluated.

The present study had several limitations. Because it was retrospective, there was potential bias regarding treatment selection and follow-up. In addition, the study sample was diverse, and there may have been regional or institutional variations. Therefore, further prospective studies of larger samples should be performed to identify the optimal treatment strategies for patients who are unresponsive to axitinib.

In conclusion, patients who have not undergone nephrectomy, those with a short duration of response to prior TKI treatment, and those in the IMDC poor risk category are more likely to experience early-PD while undergoing axitinib treatment for mRCC. Furthermore, if patients are diagnosed with early-PD after axitinib treatment, it may be preferable to choose a TKI for their subsequent treatment.

## Data Availability

The data supporting the findings of and the datasets generated and/or analyzed during this study will be made available by the corresponding author upon reasonable request.
